# Fluid–structure interaction analysis of the drop impact test for helicopter fuel tank

**DOI:** 10.1186/s40064-016-3040-5

**Published:** 2016-09-15

**Authors:** Xianfeng Yang, Zhiqiang Zhang, Jialing Yang, Yuxin Sun

**Affiliations:** Institute of Solid Mechanics, Beihang University, Beijing, 100191 People’s Republic of China

**Keywords:** Fuel tank, Fluid–structure interaction, Impact test, Arbitrary Lagrangian–Eulerian method

## Abstract

The crashworthiness of helicopter fuel tank is vital to the survivability of the passengers and structures. In order to understand and improve the crashworthiness of the soft fuel tank of helicopter during the crash, this paper investigated the dynamic behavior of the nylon woven fabric composite fuel tank striking on the ground. A fluid–structure interaction finite element model of the fuel tank based on the arbitrary Lagrangian–Eulerian method was constructed to elucidate the dynamic failure behavior. The drop impact tests were conducted to validate the accuracy of the numerical simulation. Good agreement was achieved between the experimental and numerical results of the impact force with the ground. The influences of the impact velocity, the impact angle, the thickness of the fuel tank wall and the volume fraction of water on the dynamic responses of the dropped fuel tank were studied. The results indicated that the corner of the fuel tank is the most vulnerable location during the impact with ground.

## Background

Nowadays, helicopters are widely used in military and civil fields owing to its unique vertical take-off and landing properties, excellent hover performance and low-speed characteristics. It is becoming increasingly obvious that helicopters play significant roles in transportation, medical rescue, aerial detection, and so on. However, crash is inevitable for helicopters during abnormal landing because the reaction time is too short for pilot to take proper action owing to its low flight height. Although several attempts have been made to improve helicopter crashworthiness and passenger safety level during the crash landing, the helicopter crashes are of frequent occurrence causing the injuries and fatalities of occupants. According to the statistics, approximately 15 % of the injuries and deaths in army and civilian helicopter accidents are caused by fuel ignition on account of the fuel tank failure (Giavotto et al. 1988). Consequently, the crashworthiness is the crucial concern in the design of a helicopter fuel tank to improve the survivability of aircraft occupants and structures under crash situations (Yang and Wu 2001).

Since the 1960s, the US army has issued the first military regulations (MIL-DTL-27422) that defines the performance requirements for helicopter fuel tanks to eliminate post-crash fire after an emergency landing (Harris et al. 2000). As described in the MIL-DTL-27422, the drop impact test must be conducted to check the dynamic response of the fuel tank. A report about transport airplane crash resistant fuel system was issued, funded by the Federal Aviation Administration (FAA), which described the basic research, testing, field investigations and production efforts to improve the state of crash fire protection (Robertson et al. 2002). Anghileri (1998) preliminary investigated the crashworthiness of fuel tank by means of experiments and simulations. Numerical models for the analysis of water sloshing in a fuel tank during crash was developed and verified by experimental results, which generates four different models to simulate the water inside the tank (Anghileri et al. 2005). Li et al. (2007) simulated the dynamic behaviors of dual layer fuel tank during the impact with the ground based on parallel computing, and the results indicated that, comparing with the recursive coordinate bisection (RCB) algorithm, their algorithm could run with high speed up ratio and parallel efficiency. Luo et al. (2007) analyzed the crashworthiness of fuel tank for helicopter by utilizing the finite element method (FEM) for the purpose of validating energy-absorption capabilities of the textile layer and protection frame. Kim and Kim (2014) numerically simulated crash behavior of fuel cell group of rotorcraft based on the smoothed particle hydrodynamics (SPH) method by utilizing the commercial software LS-DYNA. In addition, the analytical, numerical and experimental researches have been conducted to analyze the structural performance of water tank with energy absorbing support under dynamic pressure and blast loading in order to reduce the damage of structures (Wang and Liew 2015; Wang et al. 2015).

Apparently, the dynamic response of the dropped fuel tank is a representative fluid–structure interaction (FSI) problem, which is extremely complex owing to the strong nonlinearity and large deformation. The arbitrary Lagrangian–Eulerian (ALE) approach, containing both pure Lagrangian and pure Eulerian formulations, provides a useful tool for the FSI problems (Souli et al. 2000; Gadala and Wang 2015; Uchiyama 2001; Ma and Yan 2006). Bathe et al. (1999) studied the interaction between structure undergoing large deformation and fluid by using an ALE formulation for the fluid and a pure Lagrangian formulation for the structure. Pal et al. (1999) studied the nonlinear free surface oscillation of the liquid inside elastic containers by using a mixed Eulerian–Lagrangian approach. Cao and Jin (2010) constructed a finite element model of a container filled with water to predict the dynamic behavior, in which the container was modeled in Lagrangian coordinate, and water movement and sloshing in the container were modeled using the multi-material ALE method. Lu et al. (2009) numerically investigated the impact response of liquid-filled cylindrical shells, and the influences of geometric and physical parameters on the impact responses were analyzed by using ALE approach. Moreover, some other researches about the FSI problems have been done based on SPH method (Monaghan 1994; Naghipour et al. 2008). Campbell and Vignjevic (2012) depicted an overview of the coupled FE-SPH approach for modeling water impacting on structures and analyzed specific issues related to floating structures. Vignjevic et al. (Vignjevic et al. 2013) simulated the bird striking on engine blades with an in house SPH code which is coupled with a transient nonlinear finite element code. Although the SPH method provides an applicable approach for the FSI problems, most researches are still limited to simplifying the model because of the large amount of calculation.

An attempt is made in this paper to investigate the dynamic response of the nylon woven fabric composite fuel tank striking on the ground based on the multi-material ALE method. The contents of the paper are as follows: section “Finite element model” gives a brief description about the multi-material ALE method and the FSI problem based on penalty method. Section “Experiment and numerical simulation” describes the physical drop impact test of helicopter fuel tank from a certain height and the corresponding numerical model for the FSI problem. In section “Results and discussions”, the accuracy of the present model is verified through comparison between the experimental and numerical results. A theoretical model using the mass-spring system is developed to calculate the magnitude and duration of the impact force. The influences of the impact velocity, the impact angle, the thickness of the fuel tank wall and the volume fraction of the water on the dynamic responses of the fuel tank are discussed. Section “Conclusions” gives some conclusions about this research.

## Finite element model

### Arbitrary Lagrangian–Eulerian formulation

The ALE approach combines the advantage of pure Lagrangian method and pure Eulerian method, which brings in a referential configuration where reference coordinate is introduced in addition to the Lagrangian and Eulerian coordinates. The mesh is allowed to move, but the movement of the mesh does not necessarily coincide with the motion of the material. Thus, it is easy to trace the free surfaces and moving boundaries accurately and to conserve the regularity of the computational mesh simultaneously.

Based on the ALE description, the material derivative with respect to the reference coordinate can be depicted as follows:

1 $$\frac{{\partial f\left( {X_{i} ,t} \right)}}{\partial t} = \frac{{\partial f\left( {\xi ,t} \right)}}{\partial t}{ + }w_{i} \frac{{\partial f\left( {x_{i} ,t} \right)}}{{\partial x_{i} }}$$

where $X_{i}$ is the Lagrangian coordinate, $\xi$ the referential coordinate, $x_{i}$ the Eulerian coordinate, $v_{i}$ and $u_{i}$ are the material and mesh velocities respectively, introducing a relative velocity $w_{i}$, the governing equations in ALE method are derived as follows:

2 $${\text{Mass}}\;{\text{equation}}{:}\,\frac{\partial \rho }{\partial t}\left| {_{{_{{_{\xi } }} }} } \right. + \rho \frac{{\partial \upsilon_{i} }}{{\partial x_{i} }} + w_{i} \frac{\partial \rho }{{\partial x_{i} }} = 0$$

3 $${\text{Momentum}}\;{\text{equation}}{:}\,\rho \frac{{\partial \upsilon_{i} }}{\partial t}\left| {_{\xi } } \right. + \rho w_{i} \frac{{\partial \upsilon_{i} }}{{\partial x_{j} }} = \frac{{\partial \sigma_{ij} }}{{\partial x_{j} }} + \rho b_{i}$$

4 $${\text{Energy}}\;{\text{equation}}{:}\,\rho \frac{\partial E}{\partial t}\left| {_{\xi } } \right. + \rho w_{i} \frac{\partial E}{{\partial x_{i} }} = \sigma_{ij} \frac{{\partial v_{i} }}{{\partial x_{j} }} - \frac{{\partial q_{i} }}{{\partial x_{i} }}$$

where $\rho$, $b_{i}$, $q_{i}$ and *E* are the density, the body force, the thermal flux and the total energy, respectively. Furthermore, the constitutive equation can be described as follows:

5 $$\sigma_{ij} = - p\delta_{ij} + \tau_{ij}$$

The constitutive equation of the fluid is composed of the equation of state (EOS) and the material model to describe the fluid-like deformation characteristics with the EOS defining the volumetric compression or expansion behavior of the fluid and the material model describing the relationship between the shear stress and the shear strain rate.

### Fluid structure interaction

**Fig. 1:**
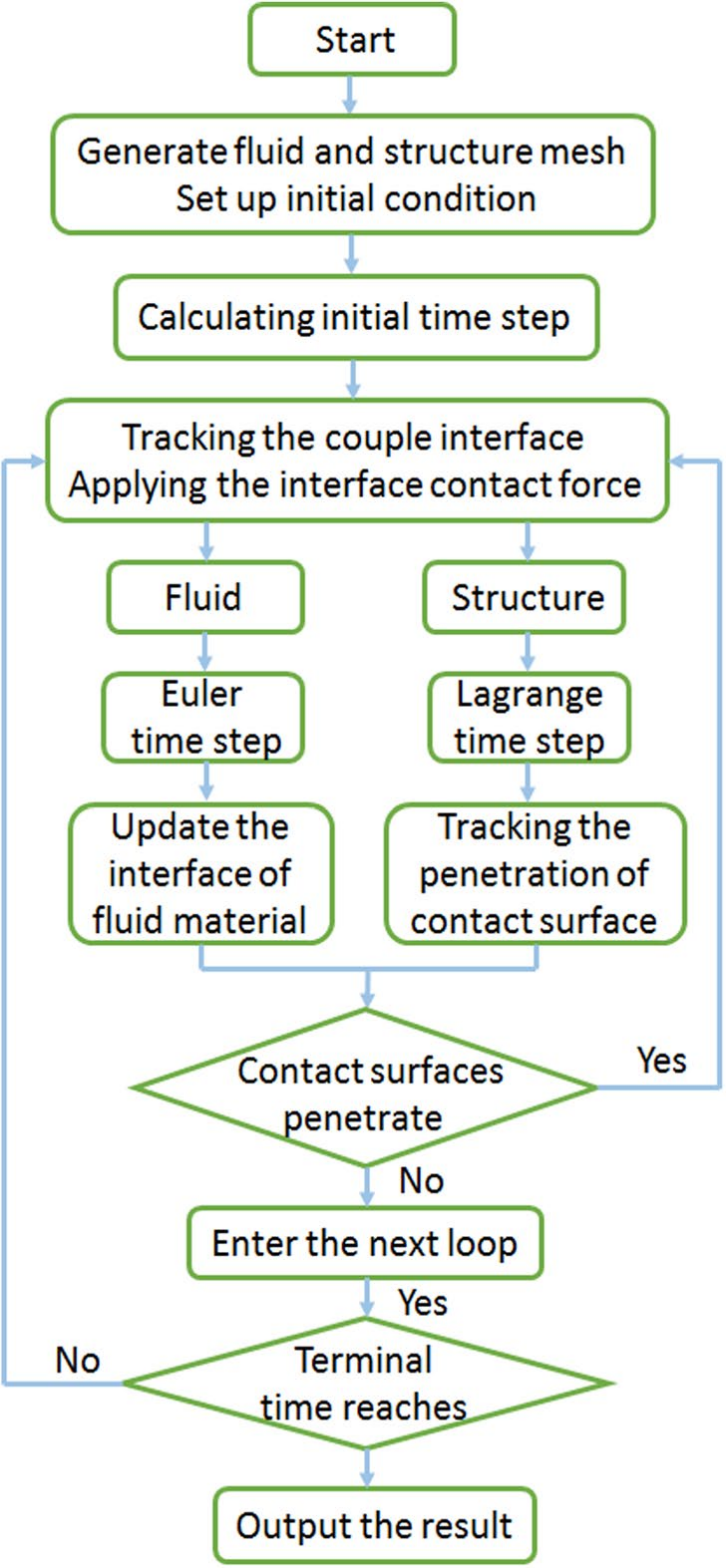
The whole procedure of the FSI problem

Fluid structure interaction is an interdisciplinary subject based on solid mechanics and fluid mechanics, in which the solid deforms or moves under the action of fluid pressure, and the solid in turn has an influence on the distribution and magnitude of the flow field. The numerical method to deal with the FSI problem includes Monolithic method and iterative method (Komatsu 1983), with the former regarding the solid and fluid as a whole system, which leads to a large amount of calculation, and the latter an effective and widely applicable method. In this paper, the iterative method is utilized and the procedure is shown in Fig. 1.

In ALE description, the deformation of the fuel tank is described by the following formulation:

6 $$\rho_{s} \frac{{\partial^{2} u}}{{\partial t^{2} }}\left| {_{{X_{i} }} = \frac{{\partial \sigma_{ij} }}{{\partial x_{i} }}} \right. + f_{i}$$

where $\rho_{s}$ is the density of the fuel tank, $f_{i}$ the body force and $u$ the displacement of the fuel tank.

On the FSI interface, the following compatible and equilibrium equations should be satisfied:

7 $$\upsilon = \frac{\partial u}{\partial t}\left| {_{{X_{i} }} } \right.$$

8 $$F_{f} + F_{s} = 0$$

where $\upsilon$ is the velocity of the structure described by the Lagrangian coordinate, $F_{f}$ and $F_{s}$ are the interaction forces acting on the FSI interfaces by fluid and solid, respectively.

The penalty method is adopted in this paper for the FSI problems in order to calculate the interaction force, in which normal interface springs are put between all penetrating nodes and the contact surface (Hallquist 2007). During calculation, each slave node is checked for penetration through the master surface. If it does penetrate, an interface force is applied between the slave node and its contact point with the magnitude proportional to the amount of penetration. This may be taken as the supplement of an interface spring.

## Experiment and numerical simulation

### Material properties

In practice, the fuel tank of an aircraft is installed beneath the floor of cabin. The soft fuel tank is composed of two layers—a crash-resistant outer layer made of textile and an oil-proof inner layer made of rubber—which are adhesively bonded together. Besides, a protective frame wraps round the tank so as to improve the crashworthiness. For simplification of analysis, we just study the drop impact performance of the soft fuel tank without the protective frame.

**Fig. 2:**
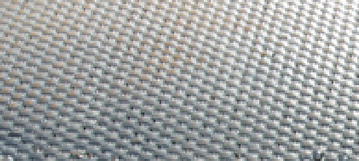
The woven material of the soft fuel tank

**Fig. 3:**
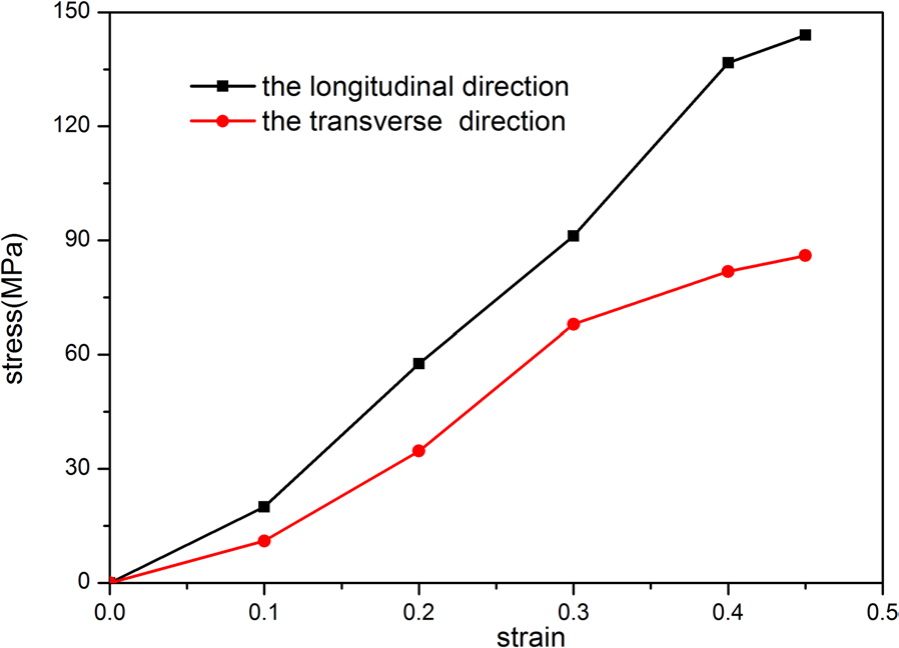
The tested stress–strain curves of the woven material in the longitudinal and transverse directions

First, uniaxial tensile tests of the woven material as shown in Fig. 2 is conducted to obtain the fundamental mechanical parameters. Since the woven material is anisotropic, the tension tests are performed separately along two orthogonal directions—longitudinal direction L and transverse direction W—according to the fiber distribution. The tested stress–strain curves of the woven material in the two directions are shown in Fig. 3. It is tested that the density of the woven material is 1150 kg/m^3^, the Young’s modulus is 1000 MPa and the Poisson’s ratio is 0.4. The average thickness of the woven material is 2 mm. It has been reported that the transverse lying fiber around the fuel tank leads to drastic deformation under water pressure, and the deformation of the longitude lying fiber is relatively small due to its unrestricted motion along with the free drop of the water (Luo et al. 2007). Thus, the woven material can be dealt as an equivalent isotropic material with the transverse lying fiber being the major parameter.

### Experimental test

In order to study the behavior of fuel tank under drop impact load and to validate the numerical model, a series of drop impact tests are conducted by means of a fuel tank filled with water. The soft tank made of only one layer of nylon woven fabric composite material is used in the experiment. There is a hole on the accessory panel located at the bottom of the fuel tank, from which water is poured into the tank. The tank has a size of 760 × 760 × 600 mm^3^, and is able to contain water of about 350 kg when fully filled.

**Fig. 4:**
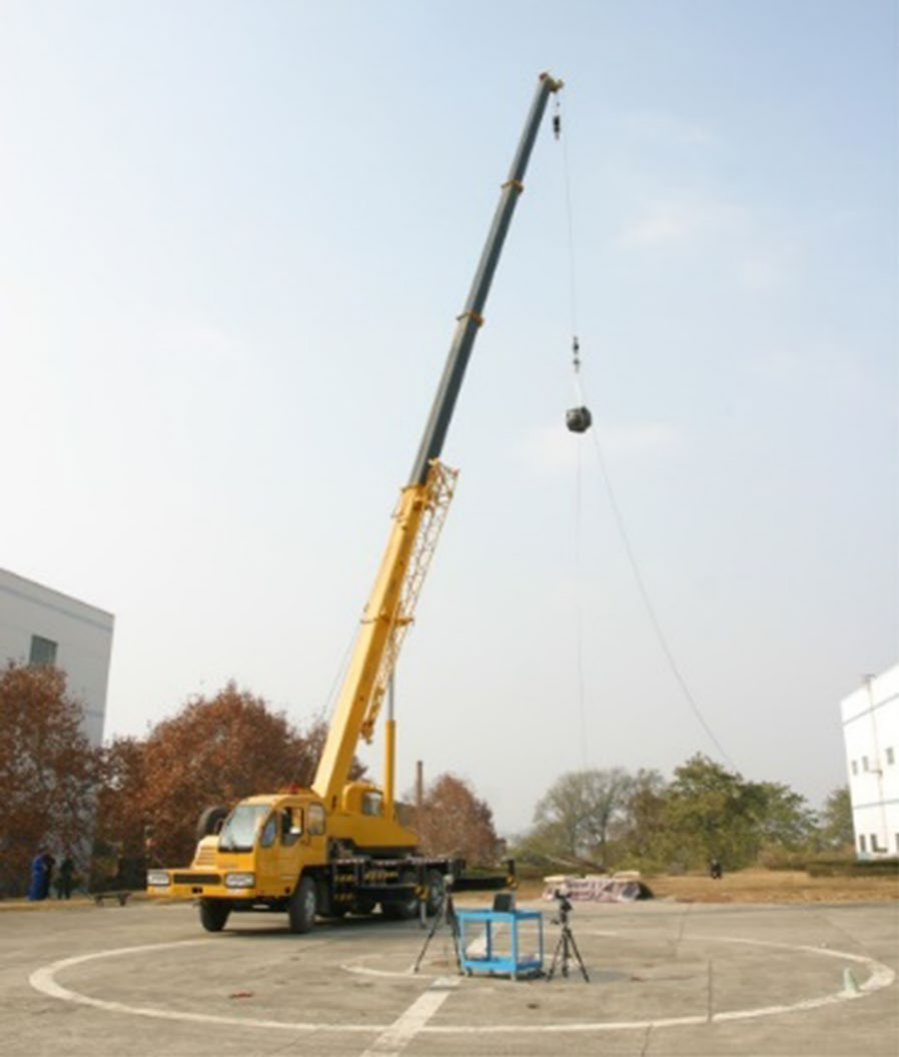
Experimental facilities of the drop impact test

**Fig. 5:**
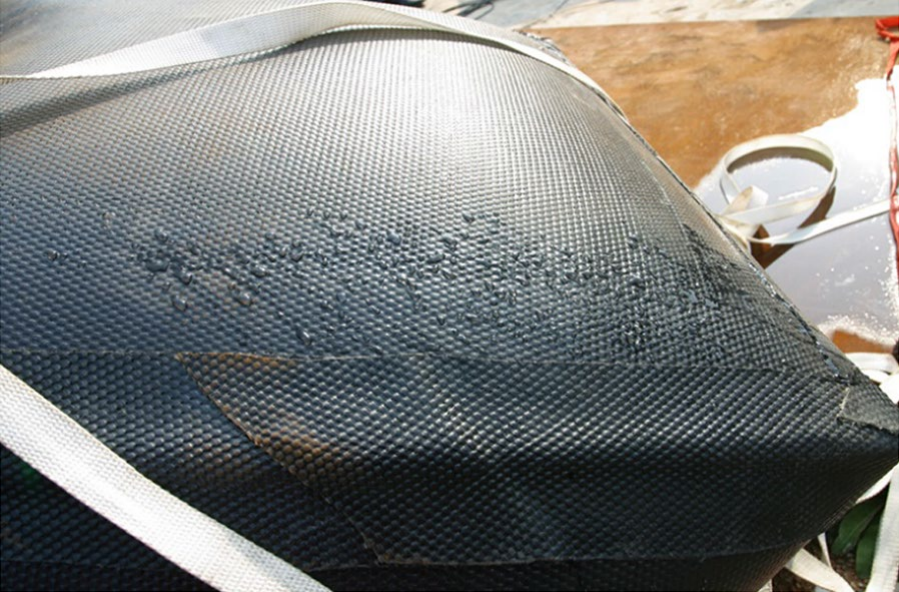
Photograph of the soft fuel tank after the drop impact test

**Fig. 6:**
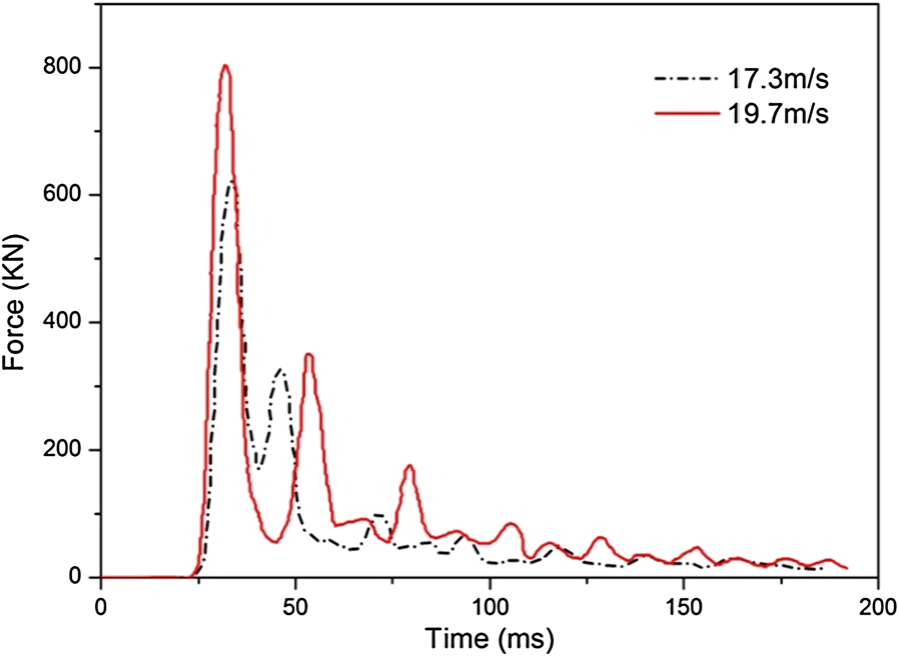
Tested time history of the impact forces under the two impact velocities

In the drop impact test, a crane is used to lift the tank to a desired height, as shown in Fig. 4, and then the tank falls freely and drops on a specialized force plate, on which four Rafah pressure sensors with the maximum force limit of 2000 kN are installed. The force plate is made of steel with the thickness of 80 mm and can be considered as a rigid body. The fuel tank is adjusted to guarantee that it drops on the force plate horizontally. In addition, a high-speed camera is adopted to capture the falling and impacting process. In the test, the tank drops from the height of 15.2 and 19.8 m and the impact velocities are 17.3 and 19.7 m/s, respectively. The photograph of the soft fuel tank after the drop impact test is shown in Fig. 5, and the tested impact force–time history curves are shown in Fig. 6.

### Numerical simulations

#### FE model

**Fig. 7:**
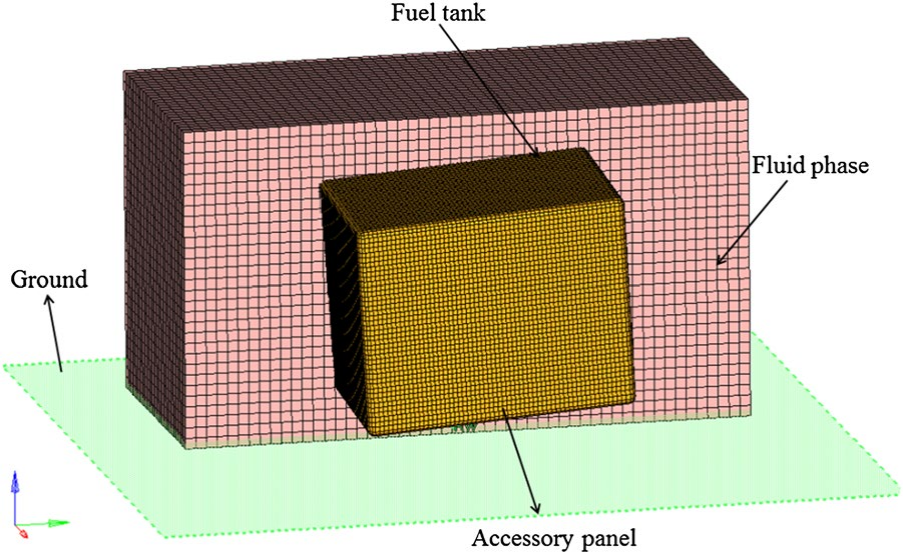
The FE model of the drop impact test

The three-dimensional FE model of the fuel tank is established according to the drop impact test and the model is composed of four parts: the soft fuel tank, the accessory panel, the ground, the fluid phase including the water inside the fuel tank and the air. The volume of the tank is 0.346 m^3^ and it is full of water. The accessory panel has the thickness of 2 mm. In the FE model, the ground is treated as a rigid body because its deformation during the impact is extremely small. Two kinds of elements are used in the model- Shell element is used for the nylon woven fabric composite material and the accessory panel, and hexahedral element is used for the fluid phase. The finite element model shown in Fig. 7 consists of 18,104 shell elements and 84,270 hexahedral elements, and totally there are 108,497 nodes.

#### Material model

The soft fuel tank is made of nylon woven fabric composites with a thickness of 2 mm and Poisson’s ratio of 0.4. The stress–strain relationship of the woven material is obtained from the tensile experiment as illustrated in Fig. 3. The material fails when the strain reaches 0.45. In practice, the tank impacts with the ground at a rather low speed, so the strain rate effect can be reasonably ignored without leading to large error. The accessory panel attached at the bottom of the tank is made of aluminum alloy. The parameters of all the materials are listed in Table 1.

**Table 1:** The material parameters

Parameter	Density (kg/m^3^)	E_m_ (MPa)	v
Fuel tank	1150	1000	0.4
Accessory panel	2700	70,000	0.33
Water	998	–	–
Air	1	–	–

With regard to the fluid material inside the fuel tank, EOS relates pressure to a specific change rate of the material volume at a physical state. The Gruneisen equation is chosen as the EOS of the water, which defines the pressure of compressed materials with cubic shock velocity-particle velocity as

9 $$p = \frac{{\rho_{0} C^{2} \mu \left[ {1 + \left( {1 - \frac{{\gamma_{0} }}{2}} \right)\mu - \frac{a}{2}\mu^{2} } \right]}}{{\left[ {1 - \left( {S_{1} - 1} \right)\mu - S_{2} \frac{{\mu^{2} }}{\mu + 1} - S_{3} \frac{{\mu^{3} }}{{\left( {\mu + 1} \right)^{2} }}} \right]^{2} }} + \left( {\gamma_{0} + a\mu } \right)E_{I}$$

where C is the speed of sound; S_1_, S_2_ and S_3_ are the coefficients of the slope of the u_s_–u_p_ curve; $\gamma_{0}$ is the Gruneisen gamma; $a$ is the first order volume correction to $\gamma_{0}$; $\mu$ is the kinematic viscosity; $E_{I}$ is the initial internal energy. Besides, the vacuum material is chosen for the air and it is a dummy material representing a vacuum in a multi-material ALE model. In the calculation, the parameters are: $C = 1650\,{\text{m}}/{\text{s}}$, $\mu = 8.684 \times 10^{ - 4}$, $S_{1} = 1.192$, $S_{2} = 0.92$, $S_{3} = 0$, $\gamma_{0} = 0.35$ and $a = 0$.

#### Boundary condition and initial condition

In the drop test, the soft fuel tank falls freely from the height of 15.8 m; however, the tank is assumed to fall from the height of 10 mm with a given initial velocity in the simulation in order to reduce the computing time. Besides, the air resistance is expressed as:

10 $$F_{D} = C_{D} \cdot \frac{1}{2}\rho S\bar{V}^{2}$$

where $C_{D}$, $\rho$, $S$, $V$ denotes the drag coefficient, air density, windward area and average velocity respectively. In this problem, $C_{D} = 0.8$, $\rho = 1.29\,{\text{kg}}/{\text{m}}^{3}$, $S = 0.5776\,{\text{m}}^{2}$, $\bar{V} = 9.85\,{\text{m}}/{\text{s}}$ (V should be the average velocity which equals to half of the final velocity) Thus, the air resistance is 28.9 N, the acceleration of the water-filled tank is 0.992 g (g is gravitational acceleration) which has a margin of error of 0.78 %. As a result, the velocity error is 0.4 %. Compared to the weight of the water-filled tank which is about 380 kg, the air resistance during the drop process is so small that it can be neglected without leading to significant error. Since the air resistance is ignored, the initial velocity of the tank is 17.3 m/s and the acceleration of gravity is 9.8 m/s^2^ along the vertical direction. It is shown from the photographs of the high-speed camera that the tank impinged on the ground with an angle less than 10°, so the tank in the FEM model is rotated about 5° before falling in order to simulate the actual case. The contact principle based on the penalty method is adopted to deal with the interaction for the FSI problem. The multi-material group is used for the problem when it’s a mixture of water and air inside the fuel tank.

## Results and discussions

### Results and analysis

**Fig. 8:**
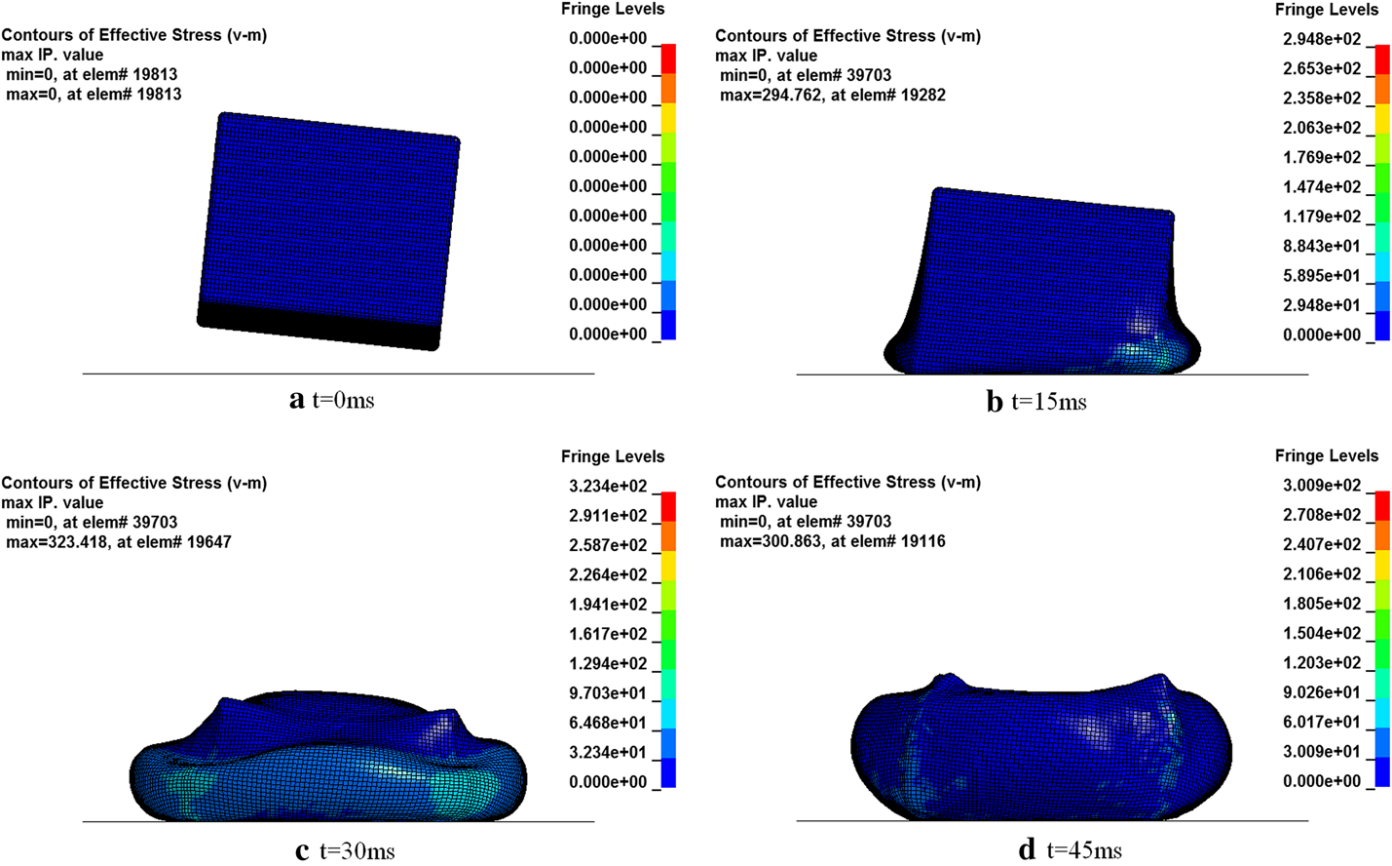
Deformation mode of the fuel tank at different times. **a** t = 0 ms, **b** t = 15 ms, **c** t = 30 ms, **d** t = 45 ms

**Fig. 9:**
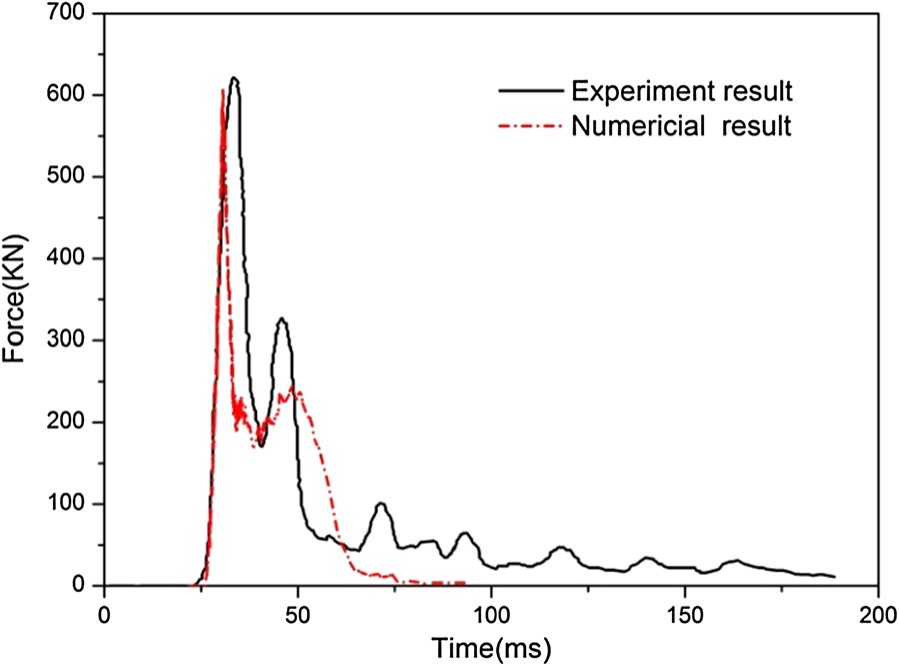
Comparison of experimental and numerical results with the impact velocity of 17.3 m/s

The calculated deformation mode of the fuel tank is shown in Fig. 8, which describes the stress distributions of the fuel tank and the corresponding configuration at different moments. The water inside the fuel tank diffuses in all directions as the fuel tank impacts with the ground and it deforms together with the soft fuel tank. It is noticeable that the fuel tank contracts inward when the container is rebounded until it terminates. Figure 9 shows the time histories of the impact force exerted on the soft fuel tank obtained from both numerical simulation and experimental results. It is shown that the two curves are very close, which proves that the result based on the numerical model is reliable. The peak force obtained from the numerical and experimental results are 607.8 and 623.9 kN, respectively, and the error is small.

Reed et al. (2000) presented a mass-spring model to analyze the drop impact behaviors of water filled containers, which regards the entire water tank system as an elastic solid body. The water-filled tank is equivalent to a mass-spring system based on the energy approach. The kinetic energy of the system is equal to the summation of the strain energies of the liquid inside the tank and that of the tank wall under the peak pressure, which means the lateral displacement reaches the maximum and the water comes to rest. According to the mass-spring model, the peak force $F_{\rm{max} }$ during the impact with the ground can be described as follows:

11 $$F_{\rm{max} } = \frac{{\pi mv_{0} }}{{\Delta t_{1/2} }}$$

12 $$\Delta t_{1/2} = \frac{\pi l}{{\sqrt {\frac{{3KE_{T} w}}{{\rho_{w} (E_{T} w + KD)}}} }}$$

where $m$ is the mass of tank filled with water, $\rho_{w}$ is density of water in tank, $\Delta t_{1/2}$ is the equivalent spring pulse time, $K$ is the bulk modulus of liquid in tank, $D$ is the diameter of the fuel tank, $E_{T}$ is the modulus of tank materials, $w$ is the wall thickness of tank, $l$ is the length of water column in container.

In Reed’s theoretical model, the reaction force is of the sinusoidal form due to the characteristic of the mass-spring system, while $\Delta t_{1/2}$ is actually the half cycle of the sinusoid. In order to assess the duration pulse time accurately, the pulse time is assumed as the duration of the sinusoidal form curves (half cycle) in the impact force curve. Thus, the maximum impact force and duration pulse time can be obtained from Eqs. (11) and (12). Table 2 shows the comparison of the duration pulse time and the peak impact force with the velocity of 17.3 m/s, which implies that the present numerical model is reliable and can be used to further study the behavior of the fuel tank under the drop impact with the ground.

**Table 2:** Comparison of experimental, numerical and theoretical results with impact velocity of 17.3 m/s

Parameter	Experimental results	Numerical results	Theoretical results
Duration pulse time (ms)	33.5	27.2	29.1
Maximum impact force (kN)	623.9	607.8	647.8

**Fig. 10:**
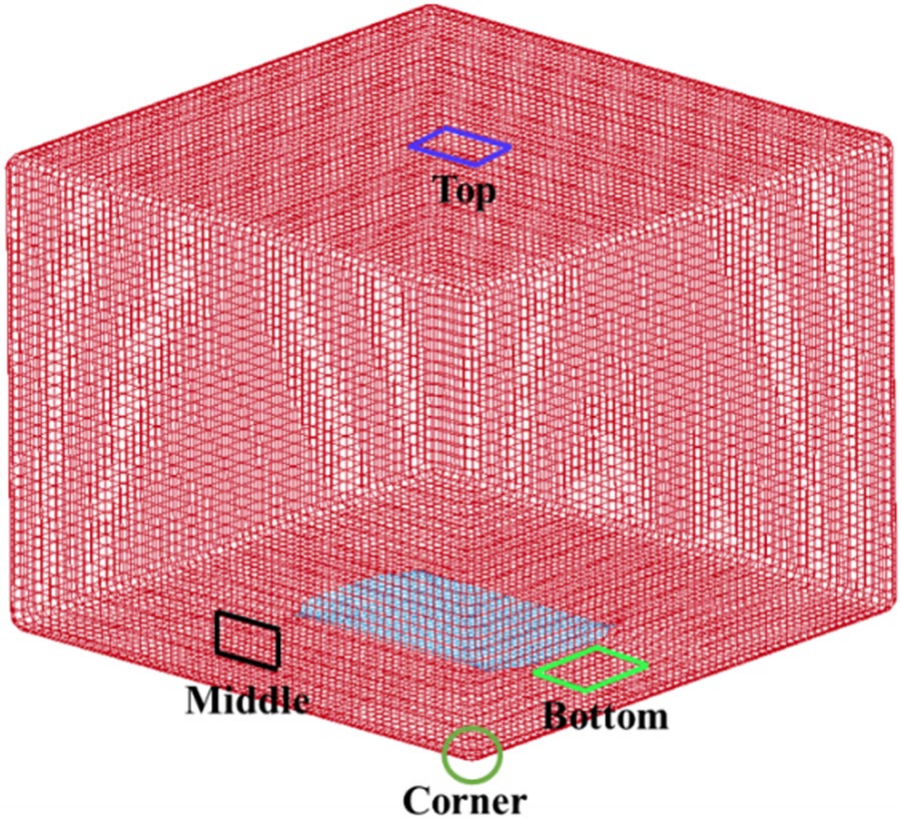
Four typical locations of the fuel tank under consideration

In the following, we take four typical locations as examples to show the distributions of the maximum principle stresses and the effective plastic strain in the fuel tank. Figure 10 illustrates the four locations: the top, the middle, the corner and the bottom of the fuel tank.

**Fig. 11:**
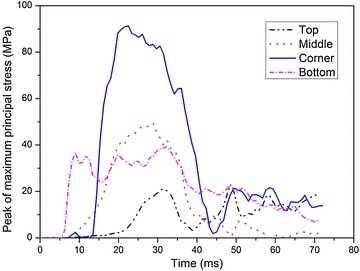
Time histories of maximum principal stresses at different locations

Figure 11 shows the time histories of the maximum principle stresses, $\sigma_{1}$, at the four locations. It is obvious that $\sigma_{1}$ arrives at the peak value in sequence from the top to the bottom of the fuel tank and then drop dramatically. When the fuel tank drops from a certain height, it impacts with the ground at the bottom at first, where $\sigma_{1}$ reaches the peak value of 39 MPa. Then the corner at the bottom of the fuel tank contacts with the ground because of the angular deflection in the air and the corresponding stress is 93 MPa, which is several times larger than that at the other locations. Soon after that, the soft fuel tank expands in all directions due to the sloshing of water inside the container and $\sigma_{1}$ is 53 MPa. Finally, the top of the fuel tank reaches its peak stress because it is the farthest from the ground.

**Fig. 12:**
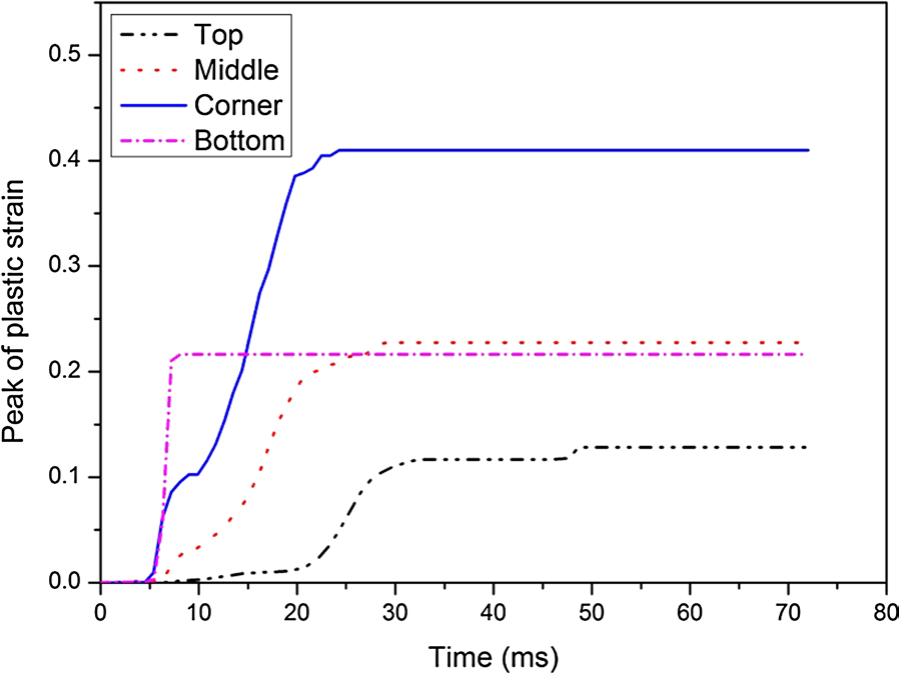
Time histories of effective plastic strain at different locations

The effective plastic strains, $\varepsilon_{\text{p}}$, at the above four locations are shown in Fig. 12. Just like $\sigma_{1}$, $\varepsilon_{\text{p}}$ reaches the peak value from the bottom to the top of the tank in sequence as well. The bottom reaches the peak plastic strain immediately when the fuel tank contacts with the ground and the corresponding strain is 0.22, then the corner arrives at the peak $\varepsilon_{\text{p}}$ of 0.41. The next peak value is 0.24 which occurs in the middle of the soft fuel tank. At last, the top of the fuel tank reaches the peak $\varepsilon_{\text{p}}$. It is shown that the maximum values of both the peak $\varepsilon_{\text{p}}$ and the peak $\sigma_{1}$ occur at the corner of the tank, where can be concluded to be the most vulnerable area during the impact with the ground.

### Parametric investigation

Since the accuracy of the numerical model has been verified through comparison with experimental results, a number of parametric studies will be performed by means of numerical simulation in this subsection in order to assess the influence of four parameters on the dynamic responses of the fuel tank: the impact velocity, the impact angle, the thickness of the fuel tank wall and the volume fraction of the water.

#### Impact velocity

**Fig. 13:**
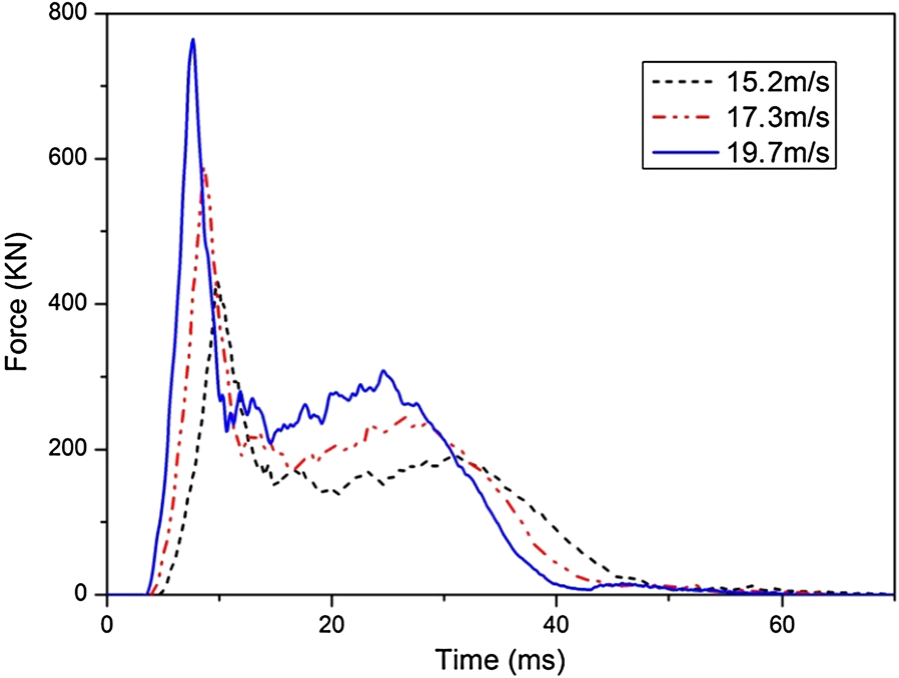
Time histories of impact forces with different impact velocities

First, the influence of impact velocity on the dynamics responses of the fuel tank is investigated. The fuel tank drops from the different heights of 11.5, 15.2 and 19.8 m, respectively. As a result, it impacts with the ground at the velocities of 15.2, 17.3 and 19.7 m/s, respectively. The tank is fully filled with water and it impacts with the ground at an angle of 5°. The impact forces acting on the tank are shown in Fig. 13. As the impact velocity increases, the impact force takes less time to reach a higher peak value.

**Fig. 14:**
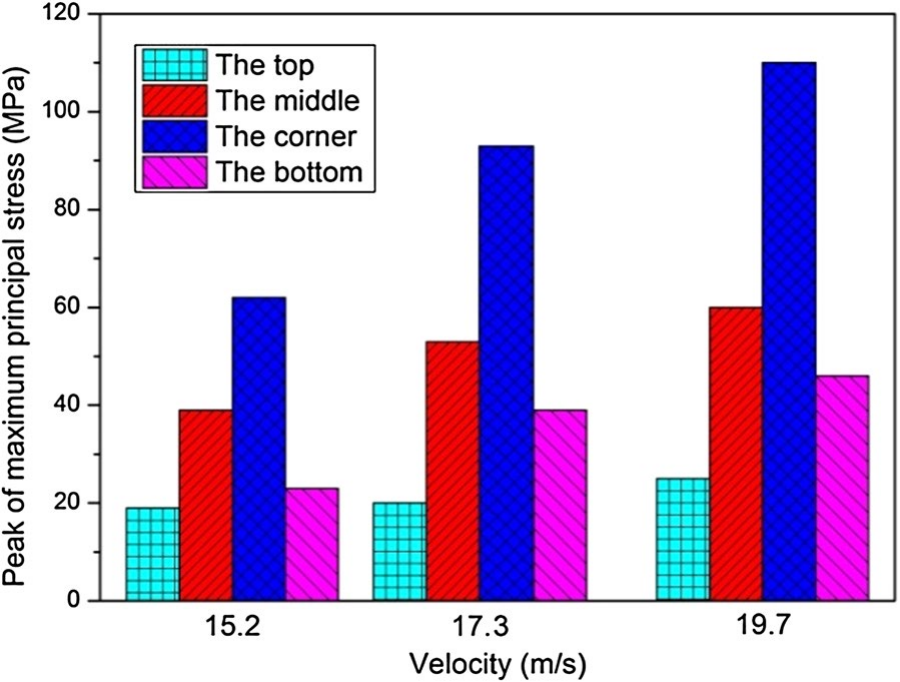
Peak of maximum principal stress in fuel tank with different impact velocity

**Fig. 15:**
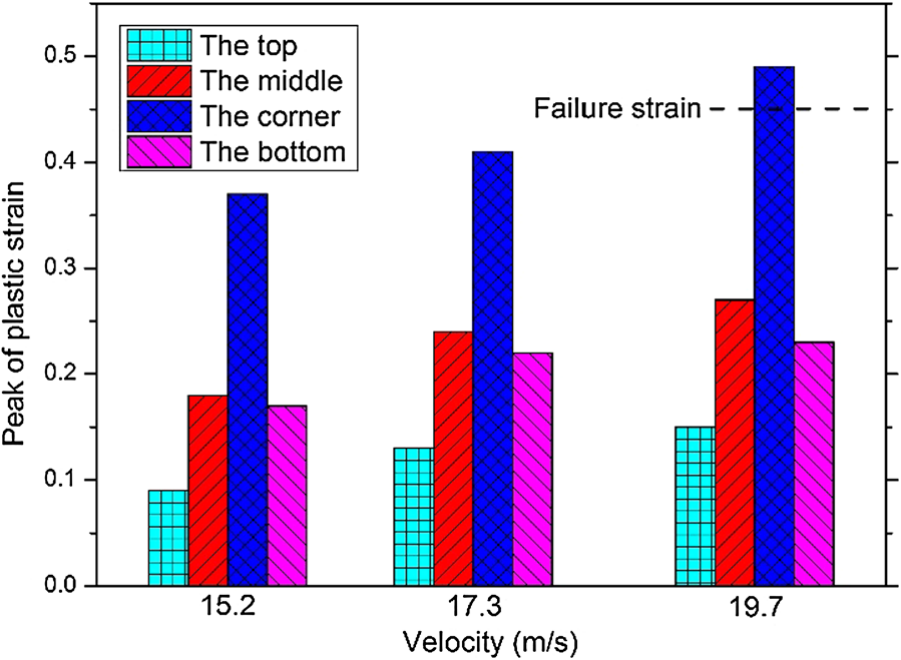
Peak of effective plastic strain in the fuel tank with different impact velocity

The peak values of $\sigma_{1}$ and $\varepsilon_{\text{p}}$ at the four locations when the impact velocities are 15.2, 17.3 and 19.7 m/s are shown in Figs. 14 and 15, respectively. It is shown in the figures that the peak values of both $\sigma_{1}$ and $\varepsilon_{\text{p}}$ increase as the impact velocity increases. The stress at the corner of the fuel tank is always the highest among the four locations. In particular, $\varepsilon_{\text{p}}$ of the corner exceeds the failure strain and breakage occurs eventually when the impact velocity is 19.7 m/s. What’s more, as the impact velocity increases, the middle area of the fuel tank is the second possible location to be destroyed during the impact.

#### Impact angle

Secondly, the influence of impact angle on the dynamic response of the fuel tank is discussed. The tank can rotate around the X and Y axes. In the simulation, four sets of rotation angles are selected: [0°,0°], [3° (around X axis), 0°], [3° (around X axis), 3° (around Y axis)] and [6° (around X axis), 0°]. The impact velocity is 17.3 m/s. The fuel tank is full of water and the wall thickness is 2 mm.

**Fig. 16:**
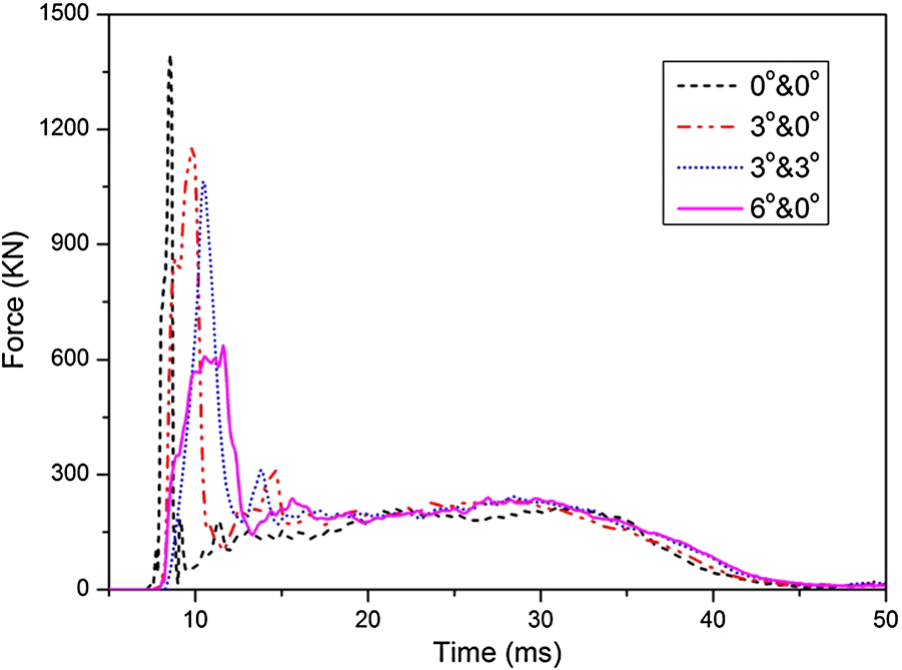
Time histories of impact forces with different impact angles

Figure 16 compares the time histories of impact force acting on the tank with different impact angles, which shows that the peak impact force decreases as the rotate angle increases. The reason might be that the effective contact area diminishes when the fuel tank rotates with a specified angle so that the tank wall has a resistance on the movement of water compared with the horizontal fuel tank. Furthermore, the peak impact force acting on the fuel tank rotating around X and Y axes simultaneously is only a little lower than that of the case rotating only around X axis.

**Fig. 17:**
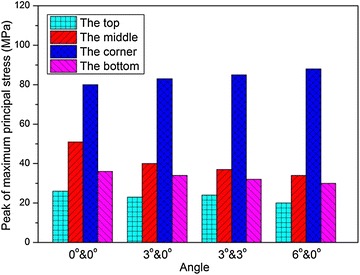
Peak of maximum principal stress with different impact angles

**Fig. 18:**
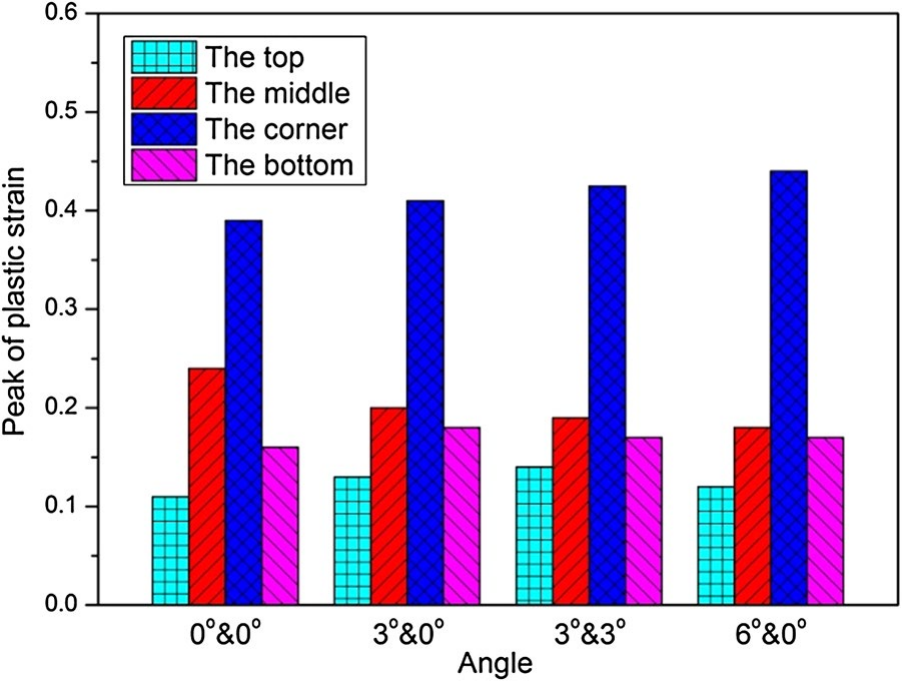
Peak of effective plastic strain with different impact angles

Figures 17 and 18 illustrate the peak values of the maximum principal stress, $\sigma_{1}$, and the effective plastic strain, $\varepsilon_{\text{p}}$, with different angles, respectively. It’s readily apparent that $\sigma_{1}$ at the corner of the fuel tank is always larger than that at the remote locations. At the corner location, the peak values of $\sigma_{1}$ and $\varepsilon_{\text{p}}$ increase as the rotating angle increases. However, at the bottom and middle of the tank, $\sigma_{1}$ decline somewhat in magnitude over the impact angle. And its tendency is not monotonic at the top of the tank.

#### Thickness of fuel tank wall

The thickness of the soft fuel tank wall is an important factor as well and it has a great influence on the dynamic behavior of the dropped fuel tank. The fuel tanks with the wall thickness of 1, 2 and 3 mm are considered in this subsection. The fuel tank fully filled with water impacts with the ground at an angle of 6° and the impact velocity is 17.3 m/s.

**Fig. 19:**
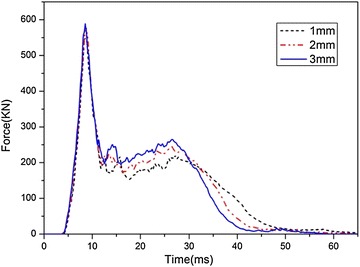
Time histories of impact forces acting on the bottom of the fuel tank with different wall thickness

The time histories of the impact forces acting on the fuel tank with different wall thickness are shown in Fig. 19. It can be seen that the peak force induced by the impact increases when the thickness of the fuel tank wall increases, as a result of the promotion of its weight.

**Fig. 20:**
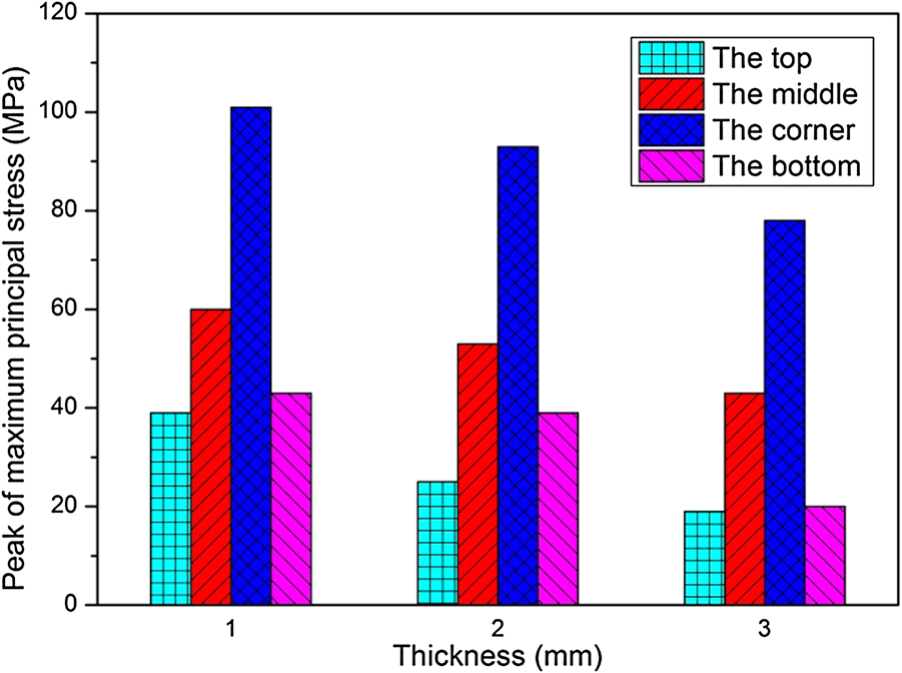
Peak of maximum principal stress with different wall thickness

**Fig. 21:**
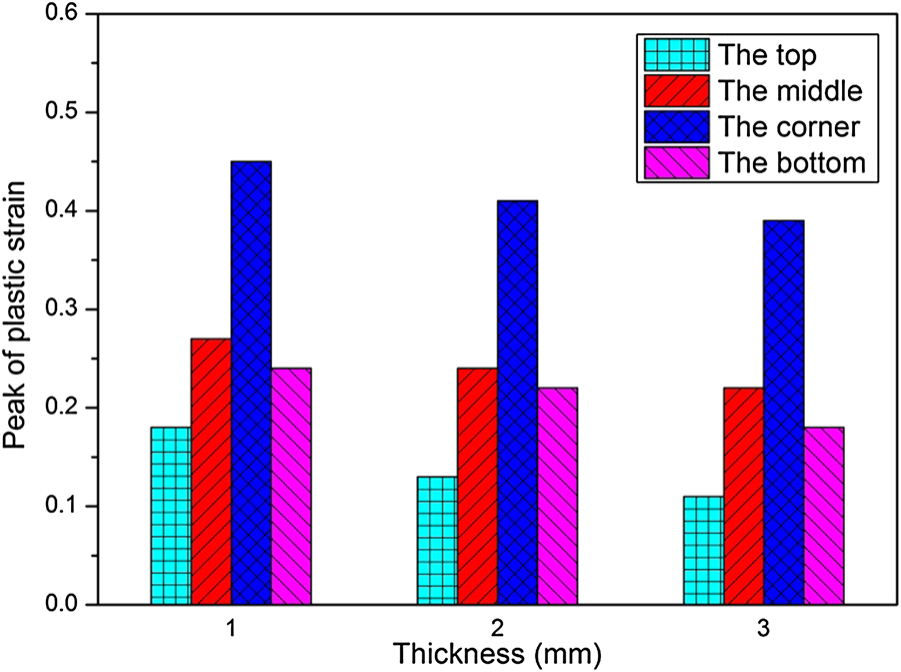
Peak of effective plastic strain with different wall thickness

In addition, the peak values of the maximum principal stress, $\sigma_{1}$, and the effective plastic strain, $\varepsilon_{\text{p}}$, induced during impact at the four locations are shown in Figs. 20 and 21, respectively. It is shown that the peak values of both $\sigma_{1}$ and $\varepsilon_{\text{p}}$ decrease as the wall thickness increases, and the tendency is identical at the four locations. Thus we can draw a conclusion that the fuel tank with thicker wall has higher strength as well as better crashworthiness during the drop impact with the ground. However, it is an inevitable issue that the tank weight also increases with the wall thickness, which results in the increasing weight of a helicopter. So it is important to find out the optimized wall thickness.

#### The volume fraction of water

Finally, the effect of the volume fraction of water on the dynamic response of the fuel tank is investigated. In the simulation, the fuel tanks are filled with water of 25, 50, 75 and 100 %, respectively. The fuel tank impacts with the ground at an angle of 5° and the impact velocity is 17.3 m/s. The wall thickness is 2 mm.

**Fig. 22:**
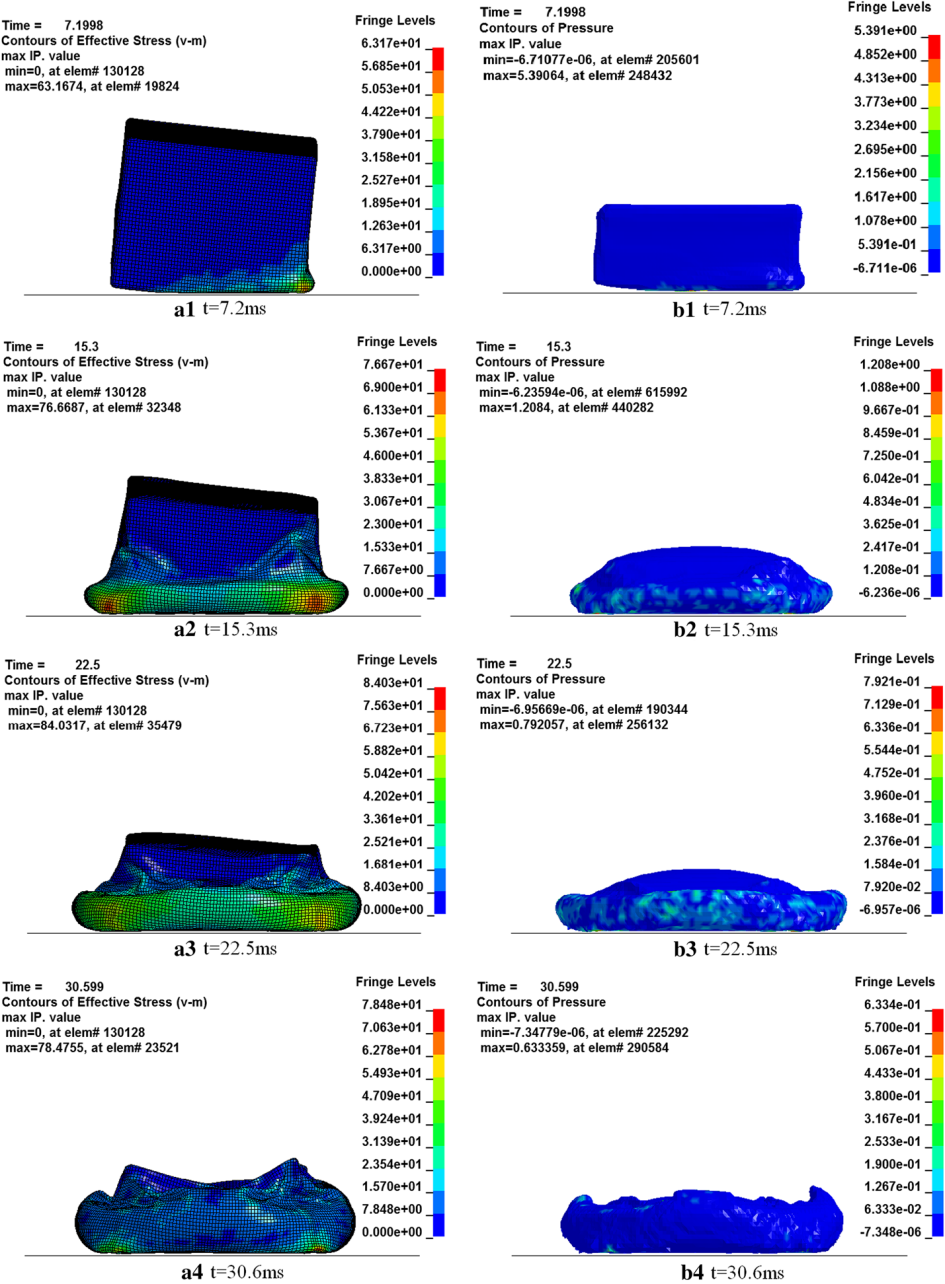
Deformation modes of the fuel tank filled with 50 % of water. **a** Stress in the fuel tank. **b** Pressure of water inside the tank

Figure 22 shows the deformation modes of the fuel tank and the inner water at several moments during the impact, with (a) showing the contours of stress in the tank and (b) the contours of water pressure inside the tank. The stress wave propagation in the tank is shown clearly. It can be seen that the Von Misses stress is high at the bottom of the fuel tank at the beginning and it propagates inwards. The failure of the fuel tank may initiate at the bottom where the von Misses stress is the highest. The flexural fold occurs in the middle of the fuel tank after the bottom entirely impact with the ground, which is because the water in the tank is half-filling. The water inside the fuel tank diffuses in all directions after the tank impact with the ground, which leads to the higher Von Misses stress around the water tank, and then the outermost water moves upward along the wall when the fuel tank extends to a limiting position.

**Fig. 23:**
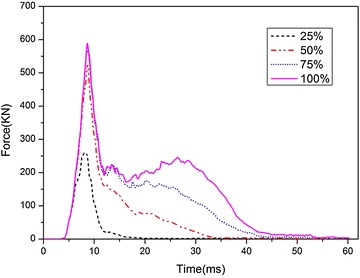
Time history of impact force with different water fraction

Figure 23 compares the time histories of impact forces acting on the tank with different water fractions. It can be seen that as the water fractions increases, the peak impact force as well as the duration time increases. However, the influence of water fraction on the peak force is little when the water exceeds half of the total volume of the tank.

**Fig. 24:**
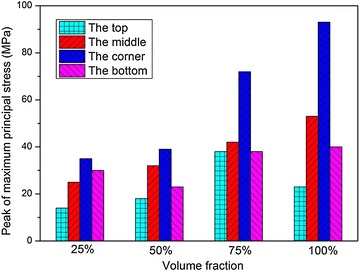
Peak of maximum principal stress with different volume fractions of water

**Fig. 25:**
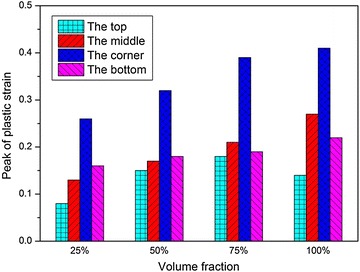
Peak of effective plastic strain with different volume fractions of water

The peak values of $\sigma_{1}$ and $\varepsilon_{\text{p}}$ at the four locations under different water fractions are illustrated in Figs. 24 and 25, respectively. Just like the load cases discussed above, $\sigma_{1}$ at the corner of the fuel tank is always larger than that at the other locations, and the peak values of $\sigma_{1}$ and $\varepsilon_{\text{p}}$ increase as the water fractions increases. As we known, the energy absorbed in the impact process increases as the water fraction increases. Therefore, as the volume fractions of the water increases, the deformation of the fuel tank may become smaller. However, the compressibility of water is negligible and the stiffness is quite high, so its shock absorption is less than that of the fuel tank. As a result, the peak value of $\sigma_{1}$ of the fuel tank is the largest when the tank is fully filled with water.

## Conclusions

The dynamic response of the fuel tank drop impact with the ground was studied in this paper. An impact drop test was conducted to study the failure behavior of the woven material used in the fuel tank. An explicit nonlinear FE model of the fuel tank was established and the multi-material ALE approach was adopted to solve the fluid structure interaction problem based on the penalty method. The numerical results were compared with experimental results, and good agreement was obtained. The influences of the impact velocity, the impact angle, the thickness of the fuel tank wall, the volume fraction of water on the dynamic responses of the fuel tank were investigated. The main findings from the numerical simulations and experimental tests are summarized as follows:

1. The multi-material ALE approach based on the penalty method was applicable to deal with the FSI problem. The numerical model presented in the paper can provide an effective approach to predict the dynamic responses of the soft fuel tank of the helicopters and can help to improve the designing of the fuel tank.
2. Both the simulation and experimental results implied that the corner of the fuel tank is the most vulnerable location during the impact process, and failure appears when the impact velocity increases to 19.7 m/s. As the impact velocity increases, the middle area of the fuel tank is the second location to be destroyed during the impact.
3. As the impact velocity or the volume fraction of water increases, or the thickness of the fuel tank wall decreases, the peak values of the impact force, the maximum principal stress and the effective plastic strain all increases. As the impact angle increases, the peak impact force decreases; however, the peak values of the maximum principal stress and the effective plastic strain increase only at the corner, but decrease at the bottom and middle of the tank, while the tendency is not monotonic at the top of the tank.

## List of symbols

$$X_{i}$$: Lagrangian coordinate
$$\xi$$: referential coordinate
$$x_{i}$$: Eulerian coordinate
$$v_{i}$$: material velocity
$$u_{i}$$: mesh velocity
$$w_{i}$$: reference velocity
$$\rho$$: material density
$\sigma_{ij}$: components of stress tensor
$$b_{i}$$: body force
$$q_{i}$$: thermal flux
$$E$$: total energy
$$\tau_{ij}$$: deviatoric stress
$$\delta_{ij}$$: stress tensor
$\rho_{s}$: density of the fuel tank
$u$: displacement of the fuel tank
$\sigma_{1}$: maximum principal stress
$F_{f}$: interaction forces acting on FSI interfaces by fluid
$S_{1} \sim S_{3}$: coefficients of the slope
$$\gamma_{0}$$: Gruneisen gamma
$C$: speed of sound
$a$: first order volume correction to $\gamma_{0}$
$$\mu$$: kinematic viscosity
$F_{\hbox{max} }$: peak force during the impact
$m$: mass of water-filled tank
$\rho_{w}$: density of water in tank
$\Delta t_{1/2}$: equivalent spring pulse time
$K$: bulk modulus of liquid in tank
$E_{T}$: modulus of tank material
$w$: wall thickness of the fuel tank
$l$: length of water column in container
$D$: diameter of the fuel tank
$v_{0}$: impact velocity of the fuel tank
$\varepsilon_{\text{p}}$: effective plastic strain
$F_{s}$: interaction forces acting on FSI interfaces by fluid solid
